# TB treatment delays and associated risk factors in Dushanbe, Tajikistan, 2019–2021

**DOI:** 10.1186/s12879-024-10265-8

**Published:** 2024-12-18

**Authors:** Radzhabali Sharifov, Dilyara Nabirova, Zulfiya Tilloeva, Sanam Zikriyarova, Nishant Kishore, Navruz Jafarov, Salomuddin Yusufi, Roberta Horth

**Affiliations:** 1 Asfendiyarov Kazakh National Medical University, Almaty, Kazakhstan; 2 Municipal Disinfection Station, Dushanbe, Tajikistan; 3 Central Asia Advanced Field Epidemiology Training Program, Almaty, Kazakhstan; 4 Central Asia Office, U.S. Centers for Disease Control and Prevention, Almaty, Kazakhstan; 5 Global Immunizations Division, U.S. Centers for Disease Control and Prevention, Atlanta, USA; 6 Ministry of Health and Social Protection of the Republic of Tajikistan, Dushanbe, Tajikistan; 7 Avicenna Tajik State Medical University, Dushanbe, Tajikistan

**Keywords:** Patient delay, Health care system delay, Tuberculosis, COVID-19, Tajikistan

## Abstract

**Background:**

In Tajikistan, where there are about 8,000 cases annually, many new cases are being diagnosed with severe disease, indicating a delay in receiving care. We aimed to estimate the proportion with delayed care and the main factors contributing to delayed care.

**Methods:**

Using a retrospective cohort design, we conducted a study that included all people aged over 15 years who were newly diagnosed with pulmonary TB in Dushanbe from 2019 to 2021. We defined ‘patient delay’ as > 14 days from TB symptom onset to the first provider visit and ‘provider delay’ as > 3 days from the first visit to treatment initiation. Data was abstracted from medical records and participants were interviewed in-person. Multivariable negative binomial regression was used to estimate adjusted risk ratios (aRR) and 95% confidence intervals (CI).

**Results:**

Of 472 participants, 49% were male, 65% had lung tissue cavitation, 33% had drug resistant TB, 11% had diabetes, 4% had HIV, and. Reported cases dropped from 196 in 2019 to 109 in 2020 and increased to 167 in 2021. The proportion of people experiencing patient delays was 82%, 72%, and 90% per year, respectively. The proportion of provider delays was 44%, 41% and 29% per year. Patient delay was associated with year (aRR: 1.09 [CI:1.02–1.18] in 2021 vs. 2019), age (aRR:0.91 [0.82–0.99] for 40–59-year-olds vs. 15–39-year-olds), having HIV (aRR:1.22 [1.08–1.38]), having blood in sputum (aRR:1.19 [1.10–1.28]), chest pain (aRR:1.32 [1.14–1.54]), having at least two structural barriers vs. none (aRR:1.52 [1.28–1.80]), having one of the following barriers: long wait lines (aRR:1.36 [1.03–1.80]), feeling that healthcare services were expensive (aRR:1.54 [1.28–1.85]), or having no time or too much work (aRR:1.54 [1.29–1.84]). Provider delay was associated with year (aRR: 0.67 [0.51–0.89] in 2021 vs. 2019), patients having to pay for X-ray services (aRR: 1.59 [1.22–2.07]) and lacking direct-observed-therapy (DOTS) in facility (aRR: 1.61 [1.03–2.52]).

**Conclusions:**

Patient delay was high before the COVID-19 pandemic and increased in 2021, while provider delay decreased during this time. Addressing structural barriers to healthcare services, such as increased DOTS facilities, expanded hours, and zero fees, may decrease delays.

**Supplementary Information:**

The online version contains supplementary material available at 10.1186/s12879-024-10265-8.

## Background

Timely diagnosis and treatment are essential interventions to eliminate tuberculosis (TB) and to reach global targets to reduce TB incidence by 90% and TB mortality by 95% by 2035 [1, 2]. Delayed TB treatment initiation not only increases the risk of severe disease and mortality but also prolongs the duration of infectiousness and contributes to ongoing community transmission [3–5].

In 2020, the first year of the COVID-19 pandemic, 1.5 million people died of TB globally [6]. This was a reversal in global trends in TB mortality, where it had reached a historical low of 1.4 million in 2019 after a decade of decline. This increase was likely influenced by many factors, including disruptions in the provision of and access to TB treatment services during the pandemic [7, 8].

In Tajikistan, substantial progress has been made in treating TB over the past decade. However, many people newly diagnosed were still being diagnosed with severe disease and mortality was high [9]. High mortality and severe disease are signs of delayed care. Delayed care can increase the spread of resistant TB, and Tajikistan has a high burden of multidrug-resistant tuberculosis (incidence: 28/100,000 in 2021) that is increasing [9, 10]. During the COVID-19 pandemic, Tajikistan experienced reversals in TB detection and treatment. For example, the number of people in treatment nationally dropped from 5,755 in 2019 to 4,148 in 2020 although the number of new cases increased from 7,700 to 8,000 in that time. Additionally, the TB mortality rate increased to 12 per 100,000 people in 2021 from a ten-year low of 8 per 100,000 people before the COVID-19 pandemic in 2019 [9].

Several modeled and observational studies globally showed that COVID-19 pandemic-associated disruptions in TB services resulted in increased delays in the diagnosis and provision of care and treatment [11–14]. Like most countries, Tajikistan also experienced disruptions in the provision of regular healthcare services during the COVID-19 pandemic, but the role these disruptions played in delayed TB care is unknown [15].

To this end, we conducted a study to estimate the prevalence of patient and provider delays in TB diagnosis and treatment and to identify factors associated with delays in Dushanbe, Tajikistan. We also aimed to determine whether COVID-19 was associated with delayed care and hypothesized that there was an association.

## Methods

We conducted a retrospective cohort study of persons aged ≥ 15 years with new laboratory-confirmed diagnosis of pulmonary TB registered during the pre-pandemic period (January 1 to December 31, 2019) and during the COVID-19 pandemic (January 1 to December 31, 2020–2021) in Dushanbe, Tajikistan. All persons registered and meeting these criteria were included in the cohort.

### Data collection

We extracted data from the national tuberculosis surveillance system OPEN MRS (open medical records system) and conducted computer-assisted face-to-face interviews. Persons were excluded if they had moved from their place of residence and could not be contacted for personal interviews or if they did not provide informed consent. Informed consent was obtained from all participants, and from parents of participants ages 15–17 years old. Data abstraction and interviews were conducted from July to August 2022. The questionnaire tool was designed based on previously published studies (Supplementary Materials).

We evaluated the accuracy and completeness of the data by comparing the data across different patient records, including the paper-based “TB Patient Notification” form, the electronic National Reference Laboratory database, and the OPEN-MRS laboratory module.

### Key variables

New laboratory-confirmed cases of pulmonary tuberculosis were defined according to the case definition used by the national TB case reporting system [16, 17]:

- New cases: patients never previously treated for TB or were on TB drugs for < 1 month.
- Pulmonary TB: TB with bacteriological confirmation or with a clinically established diagnosis with involvement of the lung parenchyma or tracheobronchial tree.
- Laboratory-confirmed TB: positive acid-fast bacilli smear, mycobacterial culture, or molecular testing (such as Xpert MTB/RIF).

Definition of delays:

- Patient delay: > 14 days from the onset of the first symptom (cough, fever, night sweats, weight loss, asthenia, and hemoptysis) to the first healthcare facility visit [18]. Those with a period of ≤ 14 days had no patient delay.
- Provider delay: Days from first contact with a healthcare facility to the start of TB treatment [19], with > 3 days selected as the cut-off for delay based on the technical equipment of TB facilities and the regulatory requirement for Tajikistan at the time [20]. Those with a period of ≤ 3 days had no provider delay.

### Statistical analysis

Data analysis was performed using R version 4.2.2 (R Foundation for Statistical Computing, Vienna). The sociodemographic and clinical characteristics of the study population were tabulated for each category within each of the study variables. Variables with discrete values are expressed as frequencies or percentages. We used the chi-square test to compare proportions across the periods of 2019, 2020, and 2021.

Because delay variables were not normal and skewed, we calculated the median and interquartile range (IQR) for each type of delay and used the Kruskal‒Wallis rank sum test to compare the length of patient and provider delays across the three years.

Independent risk factors for delays (patient and provider delays separately) were analyzed using bivariable and multivariable negative binomial regression models. Variables were included based on subject matter expertise and a literature review, taking also into consideration variables with *p* < 0.1 in bivariable analysis. We checked for multicollinearity and interaction effects. We grouped structural barrier variables into a single variable. The adjusted relative risk (aRR), confidence intervals, and p-values were calculated from multivariable negative binomial regression.

## Results

**Fig. 1:**
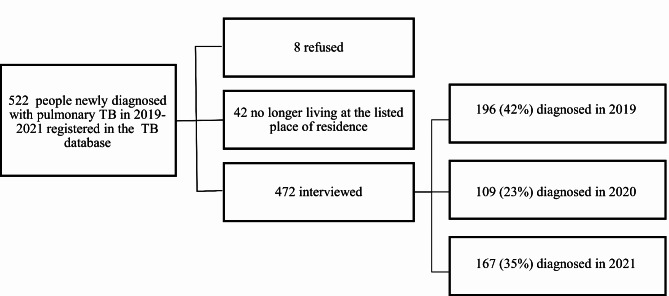
Participant recruitment diagram of people newly diagnosed with pulmonary TB, Dushanbe, 2019–2021

Of the 522 people identified as having newly diagnosed pulmonary TB from 2019 to 2021, 8 refused to participate, and 42 were no longer living in their place of residence and could not be contacted (Fig. 1). Among the 472 participants enrolled, 196 (42%) were diagnosed in 2019. There was a decrease in the number of reported new cases in 2020 to 109 (23%), and an increase to 167 (35%) in 2021.

### Characteristics of people newly diagnosed with TB

Among all participants (*n* = 472), 49% were male, 64% were married, 60% had a college or professional degree, and 15% earned less than Tajikistan Som 1000 (~$90 USD) per month (Table 1). These characteristics did not differ before and during the pandemic. A greater proportion of participants had to travel > 5 km to a healthcare facility during the pandemic than before (48% in 2020–21 vs. 36% in 2019, *p* < 0.01).

**Table 1:** Characteristics of all people newly diagnosed with pulmonary TB, Dushanbe, 2019–2021

Variables	All	2019	2020–2021	P*
	*N* (%)	*n* (%)	*n* (%)	
	*N* = 472	*n* = 196	*n* = 276	
Age in years – median [IQR]	33 [26, 51]	33 [26, 50]	34 [26, 51]	
15–39	296 (63)	124 (63)	172 (62)	0.96
40–59	107 (23)	43 (22)	64 (23)	
≥ 60	69 (15)	29 (15)	40 (14)	
Male (vs. female)	233 (49)	102 (52)	131 (47)	0.33
Marital status				
Married	303 (64)	125 (64)	178 (64)	0.78
Single	137 (29)	60 (31)	77 (28)	
Divorced or widowed	32 (7)	11 (6)	21 (8)	
Education				
Elementary or none	58 (12)	21 (11)	37 (13)	0.54
High school	132 (28)	52 (27)	80 (29)	
Professional or university	282 (60)	123 (62)	159 (58)	
Monthly HH income < TJS1000**	71 (15)	23 (12)	48 (17)	0.09
HH members numbers ≥ 5 people	121 (26)	43 (22)	78 (28)	0.12
HH > 5 km from healthcare facility	203 (43)	71 (36)	132 (48)	< 0.01
Comorbid diseases				
Cavitation of lung tissue	288 (61)	79 (40)	209 (76)	< 0.01
HIV	18 (4)	7 (4)	11 (4)	0.82
Viral hepatitis	21 (4)	6 (3)	15 (5)	0.22
Diabetes mellitus	50 (11)	13 (7)	37 (13)	0.02
Chronic nonspecific lung disease	11 (2)	8 (4)	3 (1)	0.03
Hypertension	21 (4)	7 (4)	14 (5)	0.44
Drug resistance	157 (33)	62 (32)	95 (34)	0.53
Healthcare seeking behavior				
Self-medicated	271 (57)	119 (61)	152 (55)	0.22
Visited traditional healer	30 (6)	14 (7)	16 (6)	0.56
Visited state medical facility	390 (83)	168 (86)	222 (80)	0.14
Visited private medical facility	37 (8)	15 (8)	22 (8)	0.90
Visited pharmacy	201 (43)	86 (44)	115 (42)	0.63
TB symptoms and signs				
Fatigue or weakness	439 (93)	184 (94)	255 (92)	0.53
Cough > 2 weeks	435 (92)	185 (94)	250 (91)	0.13
Night sweats	387 (82)	165 (84)	222 (80)	0.30
Chest pain	353 (75)	150 (77)	203 (74)	0.46
Fever	264 (56)	110 (56)	154 (56)	0.94
Weight loss	200 (42)	74 (38)	126 (46)	0.09
Sputum with blood	104 (22)	39 (20)	65 (24)	0.35
Dizziness	104 (22)	45 (23)	59 (21)	0.68
Patient delays***	390 (83)	161 (82)	229 (83)	0.82
Provider delays***	181 (38)	87 (44)	94 (34)	0.02

Note: Reference group for categories with no reference is not in a specified category; HH = household

* Chi-square test

** Approximately US$90 in 2023

*** Patient delay is > 14 days from first symptoms to first healthcare visit; provider delay is > 3 days from first healthcare facility visit to treatment initiation

Overall, 65% had lung tissue cavitation. The proportion with lung tissue cavitation was higher among those diagnosed during the pandemic than those diagnosed after (76% vs. 40%, respectively, *p* < 0.01). The proportion of participants with diabetes mellitus was 11% overall, and it was nearly doubled during the pandemic compared to before the pandemic (13% vs. 7%, *p* = 0.02). The proportions of participants with HIV (4% overall) or drug-resistant TB (33% overall) did not change significantly.

Neither the symptoms experienced, nor healthcare-seeking locations differed significantly between the periods. Half (57%) of the participants self-medicated, and 6% sought care from a traditional healer before seeking care at a clinic. The most common symptoms experienced were fatigue or weakness (93%), cough > 2 weeks (92%), night sweats (82%) and chest pain (75%).

The majority of participants (83%) experienced patient delays (> 14 days from the first symptom to the first healthcare visit). The proportion of participants who experienced patient delay did not differ before and after the pandemic (83% in 2019 vs. 82% in 2020–21, *p* = 0.82). Over one-third (38%) experienced provider delays (> 3 days from the first healthcare facility visit to treatment initiation). The proportion of participants who experienced provider delay was significantly greater before the COVID-19 pandemic than during the pandemic (44% in 2019 vs. 34% in 2020–21, *p* = 0.02).

### Patient and provider delays in TB care over time

**Fig. 2:**
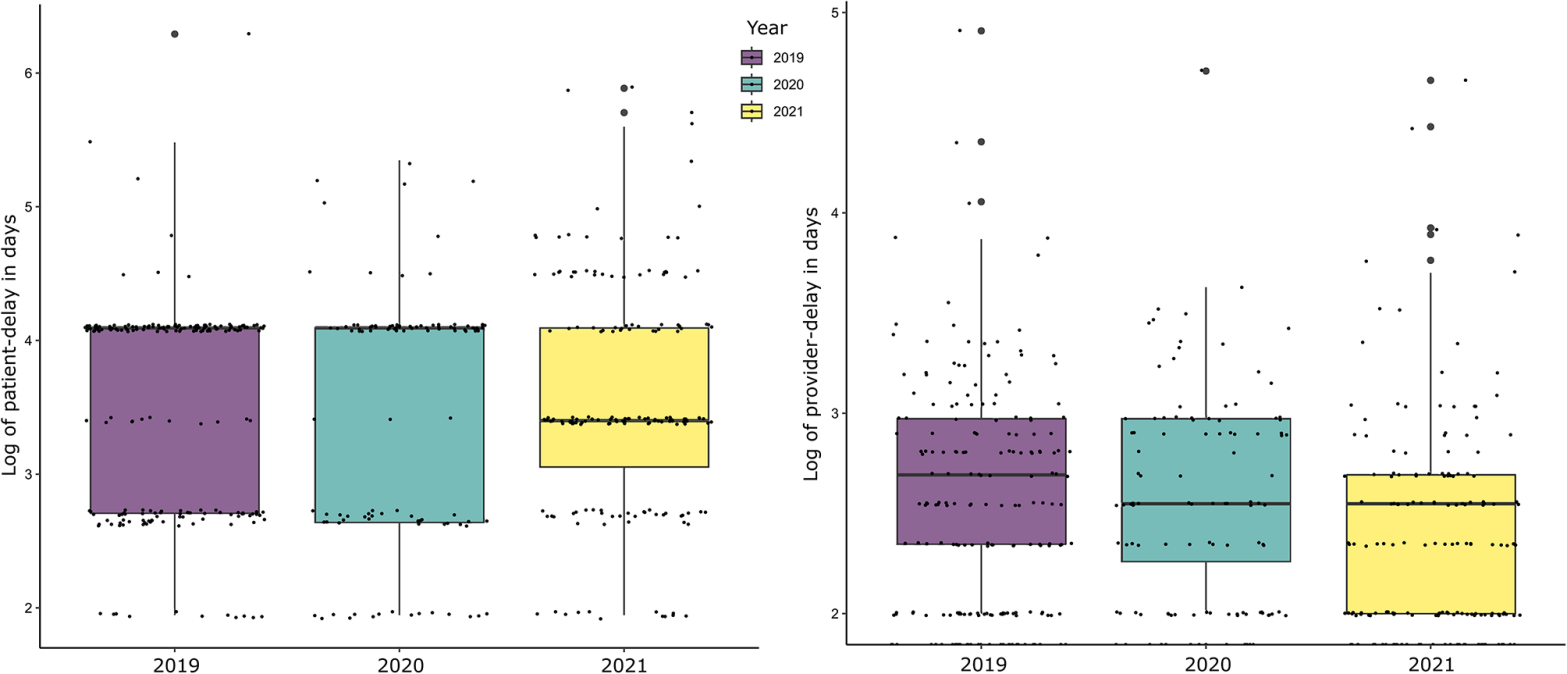
Distribution of patient and provider delays among people newly diagnosed with pulmonary TB (n=472), Dushanbe, 2019–2021 Note: Patient delay is > 14 days from the first symptoms to the first healthcare visit; provider delay is > 3 days from the initial healthcare visit to treatment initiation

The median duration of patient delay was 45 days overall (IQR: 15–60), and the distribution did not differ significantly across the three years (*p* = 0.83) (Table 2). The median provider delay was 3 days (IQR: 1–6), and the distribution differed significantly across the years from 3 (IQR: 1–6) in 2019 to 3 (IQR: 1–9) in 2020 to 2 (IQR: 1–4) in 2021 (Kruskal-Wallis rank-sum test *p* < 0.01). The distributions of patient and provider delays were similar between 2019 and 2020 and differed for 2021 (Fig. 2).

**Table 2:** Duration of days between symptom onset and first-related healthcare visit and TB treatment initiation among people newly diagnosed with pulmonary TB, Dushanbe, 2019–2021

Variables	Total^*^	2019^*^	2020^*^	2021^*^	*P* ^**^
		*n* = 196	*n* = 109	*n* = 167	
Days between TB symptom onset and 1st related healthcare visit	45 (15, 60)	60 (15, 60)	60 (15, 60)	30 (22.5, 60)	0.83
Days between 1st healthcare visit and treatment initiation	3 (1, 6)	3 (1, 6)	3 (1, 7)	2 (1, 4)	< 0.01

*Median (IQR)

**Kruskal‒Wallis rank sum test

**Fig. 3:**
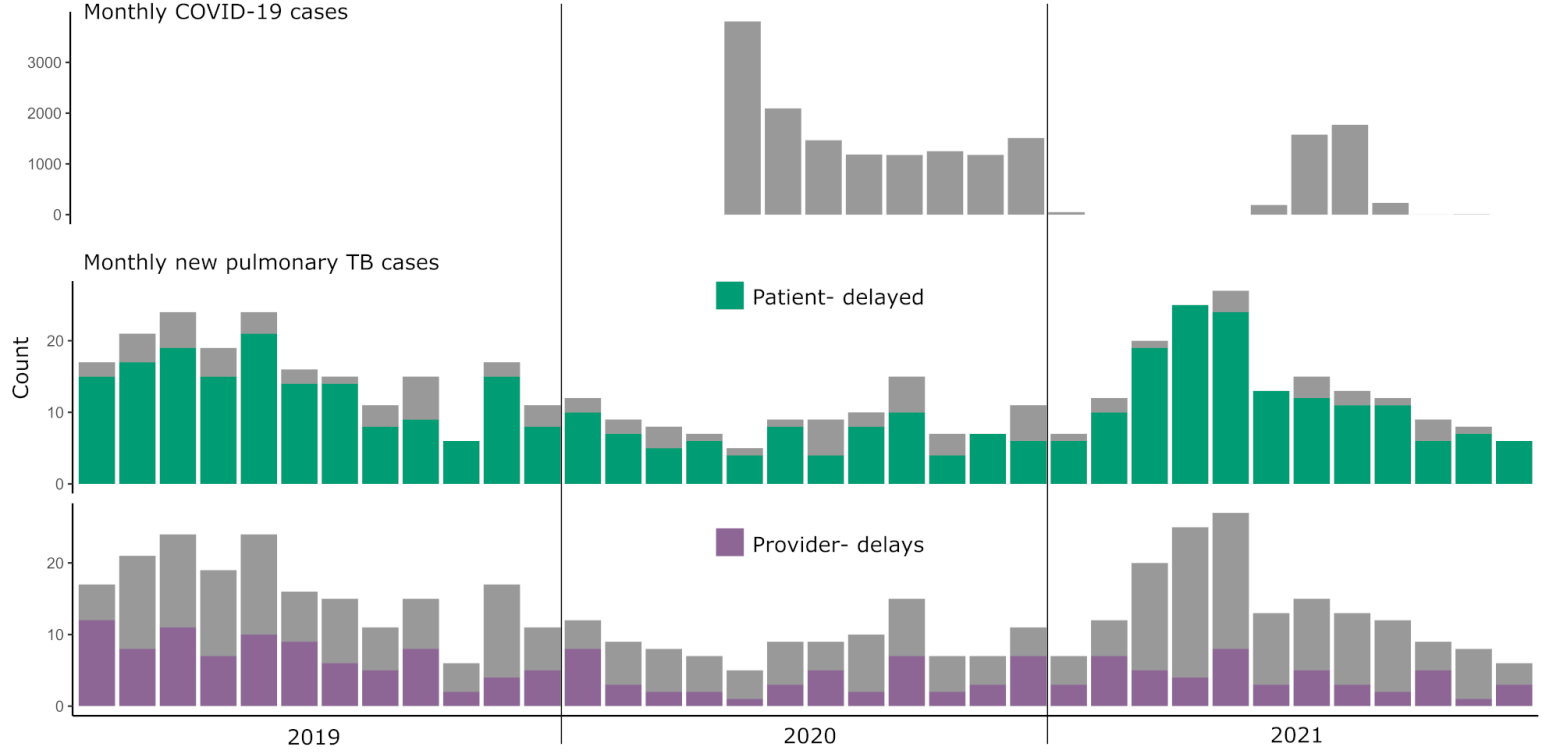
Monthly number of COVID-19 and TB cases with patient and provider delays, Dushanbe, 2019–2021 Monthly COVID-19 cases Note: Patient delay is > 14 days from the first symptoms to the first healthcare visit; provider delay is > 3 days from the first healthcare facility visit to treatment initiation

The number of people newly diagnosed with TB was lowest in May 2020, which was also the peak month of COVID-19 cases in the country; the proportion of patients diagnosed with TB was also high during these peak COVID-19 months (Fig. 3). In early 2021, when monthly COVID-19 cases were at their lowest since the pandemic inception, the number of new TB cases per month increased to above pre-pandemic levels.

### Factors associated with patient or provider delays

In multivariable analysis, the risk of patient delay was significantly greater in 2021 than in 2019 (adjusted relative risk [aRR] = 1.09, 95% CI [confidence interval]: 1.02–1.18, *p* = 0.02) (Table 3). Patient delay was associated with age and was more common among 15–39-year-olds (85% compared to 77% among 40–59-year-olds, *p* < 0.03). All people with HIV (100%) had patient delay compared to 82% among those without HIV (aRR = 1.24, 95% CI: 1.10–1.38, *p* < 0.01). The risk for patient delay was highest among those with blood in sputum (aRR = 1.22, 95% CI: 1.08–1.38, *p* < 0.01) and chest pain (aRR = 1.32, 95% CI: 1.14–1.54, *p* < 0.01). Risk was also greater for people who reported structural barriers than among those that did not have any structural barriers. Specifically, those whose only reported structural barrier was feeling that healthcare services were too expensive (aRR = 1.54, 95% CI: 1.28–1.85, *p* < 0.01); those whose only reported barrier was no time or too much work to access services (aRR = 1.54, 95% CI: 1.29–1.84, *p* < 0.01); and those who felt wait times were too long at the facility (aRR = 1.36, 95% CI: 1.03–1.80 ). Those who had at least two of those structural barriers the adjusted relative risk was 1.52 (95% CI: 1.28–1.80, *p* < 0.01) compared to those without any barriers.

**Table 3:** Factors associated with patient delay among people newly diagnosed with pulmonary TB, Dushanbe, 2019–2021

Characteristics	Patient delay^*^		RR (95% CI)	*P* ^**^	aRR (95% CI)	*P* ^***^
	No	Yes				
	*n* = 82 (%)	*n* = 390 (%)				
Pulmonary TB year of diagnosis						
2019	35 (18%)	161 (82%)	Ref.		Ref.	
2020	30 (28%)	79 (72%)	0.88 (0.77–1.01)	0.05	0.94 (0.84–1.05)	0.31
2021	17 (10%)	150 (90%)	1.09 (1.01–1.19)	**0.04**	1.09 (1.02–1.18)	**0.02**
Age in years						
15–39	45 (15%)	251 (85%)	Ref.		Ref.	
40–59	25 (23%)	82 (77%)	0.90 (0.81–1.01)	0.06	0.91 (0.83–0.99)	**0.048**
≥ 60	12 (17%)	57 (83%)	0.97 (0.87–1.10)	0.64	0.99 (0.91–1.06)	0.71
Sex						
Female	46 (19%)	193 (81%)	Ref.		Ref.	
Male	36 (15%)	197 (85%)	0.96 (0.88–1.04)	0.28	0.99 (0.91–1.06)	0.60
Have HIV						
No	82 (18%)	372 (82%)	Ref.		Ref.	
Yes	0 (0%)	18 (100%)	1.22 (1.00-1.48)	0.05	1.22 (1.08–1.38)	**< 0.01**
Had sputum with blood						
No	78 (21%)	290 (79%)	Ref.		Ref.	
Yes	4 (3.8%)	100 (96%)	1.22 (1.11–1.34)	**< 0.01**	1.22 (1.12–1.32)	**< 0.01**
Had chest pain						
No	51 (43%)	68 (57%)	Ref.		Ref.	
Yes	31 (8.8%)	322 (91%)	1.60 (1.43–1.78)	**< 0.01**	1.32 (1.14–1.54)	**< 0.01**
Had cough						
No	12 (32%)	25 (68%)	Ref.		Ref.	
Yes	70 (16%)	365 (84%)	1.24 (1.05–1.48)	**0.01**	0.91 (0.71–1.18)	0.50
Stigma barriers						
Neither	58 (34%)	111 (66%)	Ref.		Ref.	
Fear of diagnosis (only)	21 (9%)	224 (91%)	1.39 (1.24–1.56)	**< 0.01**	1.04 (0.95–1.14)	0.39
Prior bad care experience (only)	1 (14%)	6 (86%)	1.31 (0.95–1.80)	0.11	0.95 (0.76–1.18)	0.65
Both fear & bad experience	2 (4%)	49 (96%)	1.46 (1.29–1.65)	**< 0.01**	1.12 (1.01–1.25)	**0.049**
Structural barriers						
None	56 (47%)	63 (53%%)	Ref.		Ref.	
Too expensive (only)	8 (9%)	84 (91%)	1.72 (1.44–2.07)	**< 0.01**	1.54 (1.28–1.85)	**< 0.01**
No time or too much work (only)	12 (8%)	140 (92%)	1.74 (1.46–2.07)	**< 0.01**	1.54 (1.29–1.84)	**< 0.01**
Long wait times (only)	2 (3%)	62 (97%)	1.83 (1.54–2.18)	**< 0.01**	1.36 (1.03–1.80)	**0.03**
2 or more barriers	4 (10%)	36 (80%)	1.70 (1.39–2.07)	**< 0.01**	1.52 (1.28–1.80)	**< 0.01**

TB: tuberculosis; RR: relative risk; aRR: adjusted relative risk

* Patient delay > 14 days from first symptoms to first healthcare visit

** Wald P value from unconditional maximum likelihood estimation

***Z test P value from multivariable negative binomial regression

Provider delay was lower in 2021 than 2019 (29% vs. 44%) (Table 4). In multivariable analysis, 2021 was associated with reduced risk of provider delay compared to 2019 (aRR = 0.67, 95% CI: 0.51–0.89, *p* < 0.01). People who visited two or three or more healthcare facilities for their TB symptoms before their diagnosis had an increased risk of provider delay compared to those who visited only one facility (aRR = 1.64, 95% CI: 1.27–2.12, *p* < 0.01 and aRR = 1.95, 95% CI: 1.47–2.59, *p* < 0.01, respectively). Risk of provider delay was also greater among people who reported having to pay for X-rays (aRR = 1.59, 95% CI: 1.22–2.07, *p* < 0.01) compared to people who didn’t have to pay, and for people who did not have a direct observation treatment (DOT) room in their primary care facility (aRR = 1.61, 95% CI: 1.03–2.52, *p* = 0.04) compared to people who had DOT in their facility.

**Table 4:** Factors associated with provider delay among people newly diagnosed with pulmonary TB, Dushanbe, 2019–2021

Characteristics	Provider delay^*^		RR (95% CI)	*P* ^**^	aRR (95% CI)	*P* ^***^
	No	Yes				
	*n* = 291(%)	*n* = 181 (%)				
Pulmonary TB year of diagnosis						
2019	109 (56%)	87 (44%)	Ref.		Ref.	
2020	64 (59%)	45 (41%)	0.93 (0.71–1.22)	0.61	0.93 (0.72–1.20)	0.57
2021	118 (71%)	49 (29%)	0.66 (0.50–0.88)	**< 0.01**	0.67 (0.51–0.89)	**< 0.01**
Age in years						
15–39	177 (60%)	119 (40%)	Ref.		Ref.	
40–59	69 (64%)	38 (36%)	0.88 (0.66–1.18)	0.40	0.89 (0.68–1.18)	0.42
≥ 60	45 (65%)	24 (35%)	0.87 (0.61–1.23)	0.41	1.00 (0.71–1.41)	0.99
Sex						
Male	156 (65%)	83 (35%)	Ref.		Ref.	
Female	135 (58%)	98 (42%)	0.83 (0.66–1.04)	0.10	1.13 (0.90–1.41)	0.30
Number of facilities visited before diagnosis						
1	220 (67%)	106 (33%)	Ref.		Ref.	
2	53 (51%)	50 (49%)	1.49 (1.16–1.92)	**< 0.01**	1.64 (1.27–2.12)	**< 0.01**
≥ 3	18 (42%)	25 (58%)	1.79 (1.33–2.41)	**< 0.01**	1.95 (1.47–2.59)	**< 0.01**
Had to pay for X-rays						
No	124 (70%)	53 (30%)	Ref.		Ref.	
Yes	167 (57%)	128 (43%)	1.45 (1.12–1.88)	**< 0.01**	1.59 (1.22–2.07)	**< 0.01**
Had a DOT room in their primary care facility						
No	287 (63%)	172 (37%)	1.85 (1.26–2.71)	**0.03**	1.61 (1.03–2.52)	**0.04**
Yes	4 (31%)	9 (69%)	Ref.		Ref.	

TB: tuberculosis; RR: relative risk; aRR: adjusted relative risk; DOT: direct observed therapy

* Provider delay is > 3 days from the first healthcare facility visit to treatment initiation

** Wald p-value from unconditional maximum likelihood estimation

*** Z-value p-value from multivariable negative binomial regression

## Discussion

This is the first study in Tajikistan investigating the role of the COVID-19 pandemic on patient and provider delays in TB care. Patient healthcare seeking delay was high, with approximately 8 out of 10 people (83%) having experienced delayed seeking care more than 14 days after symptom onset. The median patient delay was 45 days, which is greater than the median of 29 days reported in a meta-analysis of 52 studies which included 10 high-income and 46 low-income countries [21]. Our findings are similar to those of other studies of patient delays during the COVID-19 pandemic, which reported median delays of 29 days [22], 28 days [23], and > 30 days [11]. In our study, 4 in 10 people (38%) experienced a provider delay of more than 3 days; but, the median provider delay in our study of 3 days is less than the 18-day average provider delay reported in the same meta-analysis [21].

Our study also identified a sharp decrease in the number of reported new TB cases diagnosed during the COVID-19 pandemic (from 196 in 2019 to 109 in 2020). This finding is consistent with several studies, including an 11-country study which reported a reduction of new TB cases in 2020 from 2019 [24]. While our study showed some recovery, with new TB cases in 2021 increasing to 167, case numbers did not return to pre-pandemic levels.

It is not possible to directly determine the cause for this reduction in our study. The reduction in TB cases in 2020 could be due to strict community mitigation measures implemented in the beginning of the pandemic, such as stay at home orders, closures of public places, and increased respiratory hygiene practices [25]. The periods in our study with lowest TB cases were also those with strict community mitigation in place. However, strict mitigation strategies while beneficial to reduced respiratory disease transmission also prevented people from leaving their homes to seek nonemergent care in Tajikistan and many countries. Moreover, access to nonemergent care was reduced during the early period of the pandemic in 2020 because the healthcare system was overloaded, and services were diverted to COVID-19 care.

We observed a sharp rebound in new TB cases in early 2021 when there were no or few COVID-19 cases. The 2021 surge is also consistent with global TB surveillance trends in data reported to the World Health Organization, which show that testing and treatment recovered in 2021 and reached pre-pandemic levels by 2023 [10]. Although the total number of new cases decreased in 2020, patient delay was not observed that year. This is contrary to what was expected because delays during the COVID-19 pandemic have been well documented in the literature [11, 22, 26]. While we did not observe a delay in 2020, we did observe that the proportion of people newly diagnosed with TB experiencing delays was elevated in the early months of 2021, a period when COVID-19 cases were at their lowest, and the number of new TB diagnoses was at its peak. This is likely due to an influx of people who sought care after having postponed or been unable to receive care in 2020.

Structural barriers were identified as key factors for delayed care, both for patient delay and provider delay. For provider delay, the structural barriers included the lack of DOTS services at primary health clinics, having to visit multiple clinics, and fees for X-rays. In Tajikistan, all residents are assigned to a primary health clinic. This is their first point of contact for all healthcare services. TB diagnostic tests and x-rays are provided free of charge at all public primary health clinics, but not all clinics have DOTS [27]. People who are diagnosed with TB in a primary clinic that does not offer DOTS, are referred to a clinic with DOTS, and the referral process can result in delays in initiation of treatment. Tajikistan is also a low-resource country, and primary health clinics can sometimes have shortages of TB diagnostic equipment or tests [27]. People sometimes might have to seek care in a different clinic and this would result in delayed care. Clinics will charge official and unofficial out-of-pocked fees for services and tests. Out-of-pocket payments accounted for 71.2% of health spending in Tajikistan. People unable to charge those fees might have delayed care until they can afford the fees [27]. This would explain increased provider delay among people in our study who reported having to pay a fee.

The structural barriers associated with patient delay included long wait times at clinics, feeling that services are too expensive, and having no time or too much work to be able to access services. People who had at least two of these had 50% greater risk than those who had none of these structural barriers. Other studies have reported similar structural barriers as key drivers of delayed TB care-seeking behaviors [28, 29]. These barriers are poverty-driven, and Tajikistan is among the world’s poorest countries where 2 in 10 people live below the poverty line [27]. Stigma was another important barrier. People who had both a prior bad experience with the healthcare system and a fear of diagnosis had increased risk of patient delay. Additionally patient delay was highest among people living with HIV and people that had sputum in their blood. This could be another indication that stigma is contributing to delayed care-seeking. Stigma coupled with poverty are well-documented as barriers to TB care in the global literature [30]. In Tajikistan, stigma against TB and HIV is prevalent and has been cited as being a barrier to TB treatment in a qualitative study [31].

This study is subject to several important limitations. First, our study included only cases of TB that were diagnosed and registered and would, therefore, underestimate the true incidence of pulmonary TB cases in Tajikistan. Tuberculosis is a known risk factor for COVID-19 mortality, and persons with undiagnosed TB who had or died from COVID-19 were not included in our study. Second, healthcare and public health systems were overwhelmed in the first year of the pandemic, and there may have been systematic errors in case registrations during this time. To reduce the impact of systematic errors, the study team cross-checked the data using electronic and paper-based records. Finally, the study design could be strengthened in the future by using a negative control period (e.g., years before 2019) to better control for the variability of TB diagnosis in Tajikistan over time. However, due to concerns about the quality of the data and participant recall bias, the investigators decided not to include years prior to 2019 in the study.

## Conclusions

Patient delay was high before the COVID-19 pandemic and increased in 2021. Provider delay did not increase during the pandemic years. Structural barriers, including transportation, fees, and perceived person-time costs, were important factors associated with patient and provider delays. Efforts that target these structural barriers, such as eliminating fees for diagnostic services, reducing wait times, and increasing the number of primary care clinics with directed observed treatment centers, may help reduce delays.

## Electronic supplementary material

Below is the link to the electronic supplementary material.

Supplementary Material 1

## Data Availability

The datasets used and/or analyzed during the current study are available from the corresponding author on reasonable request.

## Abbreviations

aRR: Adjusted relative risk
COVID-19: Coronavirus disease 2019
DOTS: Direct-observed-therapy
MDR: Multi-drug resistant
TB: Tuberculosis
