# Supplementary material for: TB treatment delays and associated risk factors in Dushanbe, Tajikistan, 2019–2021
Source: BMC Infect Dis. 2024 Dec 18;24:1398. doi: 10.1186/s12879-024-10265-8 (PMC11653938; doi:10.1186/s12879-024-10265-8)
Supplement: Supplementary file 1 — Supplementary Material 1 [file 12879_2024_10265_MOESM1_ESM.pdf]

ODK

1. Questionnaire No.:

____

2. Date of completion /_/_ (dd/mm/yyyy).

yyyy-mm-dd

3. Interviewer's name:

- ○
- ○
- ○

Demographics

4. Respondent ID:

____

5. Last name

____

6. Name

____

7. Date of birth:

yyyy-mm-dd

8. Gender:

- ○ Male
- ○ Female

9. Locations

latitude (x.y °)

longitude (x.y °)

altitude (m)

accuracy (m)

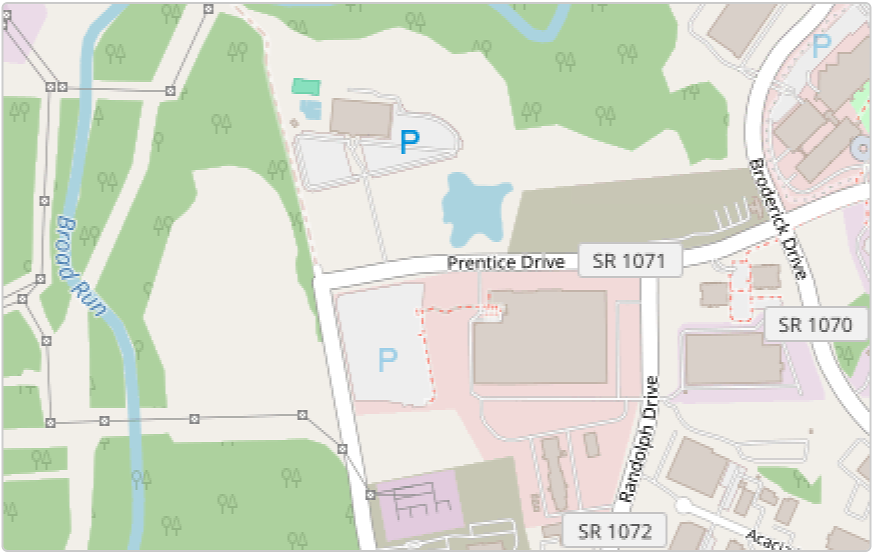

10. Phone number

11. Marital status:

- ◯ Married
- ◯ Single
- ◯ Divorced (a)
- ◯ Widow/Widower

12. Number of people living in the household with you 00 people:

13. Total family income per month

14. Education:

- ◯ Primary
- ◯ Non-primary
- ◯ Secondary vocational
- ◯ Higher
- ◯ No education

15. Employment:

- ◯ Employed
- ◯ Unemployed
- ◯ Retired
- ◯ Schoolboy/Student

15a. If you work, where:

____

16. Residence (place of residence):

- ◯ Urban
- ◯ Rural
- ◯ Homeless

Access to tuberculosis diagnostics

17. How far is your home from a primary health care facility? (distance in kilometers)

____

18. How far is your home from a primary health care facility? (time in minutes)

____

19. Is there another primary health care facility closer to your home?

- ◯ Yes
- ◯ No
- ◯ Don't know

20. Is it easy for you to get to a primary health care facility?

- ◯ Yes
- ◯ No

20a. If not, how do you get to the medical facility?

____

20b. How much money do you spend for 1 visit to a primary care facility?

____

21. Do you need to pay extra for visiting a doctor?

- Yes
- No

If yes, how much?

____

If yes, how often?

- Every visit
- Sometimes

22. Do you need to pay for TB laboratory diagnostics?

- Yes
- No

If yes, how much?

____

If yes, how often?

- Every visit
- Sometimes

23. Do you need to pay for an X-ray examination?

- Yes
- No

If yes, how much?

____

If yes, how often?

- Every visit
- Sometimes

24. Do you need to pay for additional laboratory tests (viral hepatitis, HIV test, blood biochemistry and other laboratory tests)?

- Yes
- No

If yes, how much?

____

If yes, how often?

- ◯ Every visit
- ◯ Sometimes

25. Are you satisfied with the schedule and operating hours of primary health care institutions?

- ◯ Yes
- ◯ No

Assessing factors leading to delays

» 26. Where did you first turn for medical help when you got sick?

26a. Self-medicated

- ◯ Yes
- ◯ No

26b. Turned to a traditional healer

- ◯ Yes
- ◯ No

26c. Contacted a government medical facility

- ◯ Yes
- ◯ No

26d. Contacted a private medical institution

- ◯ Yes
- ◯ No

26e. Contacted the pharmacist/pharmacy salesperson

- ◯ Yes
- ◯ No

Other:

____

27. How long were you sick before you first sought medical help?

- ◯ 1-7 days
- ◯ 8-14 days
- ◯ 15-29 days
- ◯ 1-2 months
- ◯ 3-4 months
- ◯ 5-6 months
- ◯ more than 6 months

» 28. What were the first symptoms you had to go to a primary health care facility?

28a. Cough for more than 3 weeks

- ◯ Yes
- ◯ No

28b. Sputum with blood

- ◯ Yes
- ◯ No

28c. Fever

- ◯ Yes
- ◯ No

28d. Weight loss

- ◯ Yes
- ◯ No

28e. Fatigue/weakness

- ◯ Yes
- ◯ No

28f. Dizziness

- ◯ Yes
- ◯ No

28g. Chest pain

- ◯ Yes
- ◯ No

28h. Night sweats

- ◯ Yes
- ◯ No

Other

___

29. Do you think that you put off seeking help from a medical institution for a long time?

- ◯ Yes
- ◯ No

» 30. What reasons could make you postpone seeking medical help?

30a. The symptoms are not serious and go away on their own

Possible suspected cause of delay

- ◯ Yes
- ◯ No
- ◯ I don't remember

30b. Medical facilities were too far away

Health facility means PHC facility, public or private hospital and TB facility.

- ◯ Yes
- ◯ No
- ◯ I don't remember

30c. The medical institution refused to provide assistance

Possible suspected reason for delay

- ◯ Yes
- ◯ No
- ◯ I don't remember

30d. Too many people/long waiting time

Possible expected reason for delay

- ◯ Yes
- ◯ No
- ◯ I don't remember

30e. Fear of a TB diagnosis/ Fear of what the diagnosis will reveal

Possible expected reason for delay

- Yes
- No
- I don't remember

30f. Previous bad experience (treatment failure)

Possible perceived reason for delay

- Yes
- No
- I don't remember

30g. Fear of social isolation

Possible presumptive reason for delay

- Yes
- No
- I don't remember

30h. Fear of being diagnosed with tuberculosis

Possible anticipated cause of delay

- Yes
- No
- I don't remember

30i. Fear of rejection/losing your job

Possible anticipated cause of delay

- Yes
- No
- I don't remember

30j. Lack of money to pay for treatment

Possible anticipated cause of delay

- Yes
- No
- I don't remember

30k. Difficult access to the medical center/transportation problems

Possible anticipated cause of delay

- Yes
- No
- I don't remember

30l. Poor attitude of staff towards patients

Possible anticipated cause of delay

- ◯ Yes
- ◯ No
- ◯ I don't remember

30m. Poor medical care

Possible anticipated cause of delay

- ◯ Yes
- ◯ No
- ◯ I don't remember

30n. Isolation related to COVID-19

Possible anticipated cause of delay

- ◯ Yes
- ◯ No
- ◯ I don't remember

30o. Fear of an HIV test

Possible anticipated cause of delay

- ◯ Yes
- ◯ No
- ◯ I don't remember

30p. I didn't know if that kind of help was available there

Possible anticipated cause of delay

- ◯ Yes
- ◯ No
- ◯ I don't remember

30q. I thought it was too expensive

Possible anticipated cause of delay

- ◯ Yes
- ◯ No
- ◯ I don't remember

30r. My religious beliefs did not allow me to go to a primary health care facility for help.

Possible anticipated cause of delay

- ◯ Yes
- ◯ No
- ◯ I don't remember

30s. There was no time / a lot of work

Possible anticipated cause of delay

- ◯ Yes
- ◯ No
- ◯ I don't remember

Other

____

31. Did you take any treatment before you were diagnosed with TB?

- ◯ Yes
- ◯ No

31a. If yes, which one?

____

32. How many times did you visit a medical facility before you received a final diagnosis?

____

33. Do you think that the start of tuberculosis treatment was delayed after you were diagnosed with tuberculosis?

- ◯ Yes
- ◯ No

» 34. What do you think is the reason for the delay in starting tuberculosis treatment?

34a. Fear of long-term treatment

- ◯ Yes
- ◯ No
- ◯ I don't remember

34b. Lack of anti-tuberculosis drugs in the institution

- ◯ Yes
- ◯ No
- ◯ I don't remember

34c. The tuberculosis clinic was closed

- ◯ Yes
- ◯ No
- ◯ I don't remember

34d. Lack of DOT rooms in primary health care institutions

- ◯ Yes
- ◯ No
- ◯ I don't remember

34e. Refusal to provide assistance from a medical institution

- ◯ Yes
- ◯ No
- ◯ I don't remember

34f. Distance of place of residence from medical facility

- ◯ Yes
- ◯ No
- ◯ I don't remember

34g. High cost of treatment

- ◯ Yes
- ◯ No
- ◯ I don't remember

34h. Duration of TB diagnosis

- ◯ Yes
- ◯ No
- ◯ I don't remember

34i. Fear of contracting COVID-19

- ◯ Yes
- ◯ No
- ◯ I don't remember

34j. The facility did not have beds for TB patients

- ◯ Yes
- ◯ No
- ◯ I don't remember

Other

____

» 35. How, in your opinion, can we better reduce the time required for diagnosis and treatment of tuberculosis?

35a. Timely access to a medical facility

- ◯ Yes
- ◯ No

35b. Upgrading the qualifications of health workers

- ◯ Yes
- ◯ No

35c. Organization of laboratory and diagnostic equipment in primary health care institutions

- ◯ Yes
- ◯ No

35d. Free TB diagnostic and treatment services

- ◯ Yes
- ◯ No

35e. Strengthening patronage from employees of TB Centers and DOTS offices

- ◯ Yes
- ◯ No

Respondent's comments:

____

Interviewer's comments:

____
